# Seasonal Variation in Parental Care Drives Sex-Specific Foraging by a Monomorphic Seabird

**DOI:** 10.1371/journal.pone.0141190

**Published:** 2015-11-17

**Authors:** Chantelle M. Burke, William A. Montevecchi, Paul M. Regular

**Affiliations:** Cognitive and Behavioural Ecology Program, Psychology Department, Memorial University, St. John’s, Newfoundland and Labrador, Canada; Hawaii Pacific University, UNITED STATES

## Abstract

Evidence of sex-specific foraging in monomorphic seabirds is increasing though the underlying mechanisms remain poorly understood. We investigate differential parental care as a mechanism for sex-specific foraging in monomorphic Common Murres (*Uria aalge*), where the male parent alone provisions the chick after colony departure. Using a combination of geolocation-immersion loggers and stable isotopes, we assess two hypotheses: the reproductive role specialization hypothesis and the energetic constraint hypothesis. We compare the foraging behavior of females (n = 15) and males (n = 9) during bi-parental at the colony, post-fledging male-only parental care and winter when parental care is absent. As predicted by the reproductive role specialization hypothesis, we found evidence of sex-specific foraging during post-fledging only, the stage with the greatest divergence in parental care roles. Single-parenting males spent almost twice as much time diving per day and foraged at lower quality prey patches relative to independent females. This implies a potential energetic constraint for males during the estimated 62.8 ± 8.9 days of offspring dependence at sea. Contrary to the predictions of the energetic constraint hypothesis, we found no evidence of sex-specific foraging during biparental care, suggesting that male parents did not forage for their own benefit before colony departure in anticipation of post-fledging energy constraints. We hypothesize that unpredictable prey conditions at Newfoundland colonies in recent years may limit male parental ability to allocate additional time and energy to self-feeding during biparental care, without compromising chick survival. Our findings support differential parental care as a mechanism for sex-specific foraging in monomorphic murres, and highlight the need to consider ecological context in the interpretation of sex-specific foraging behavior.

## Introduction

Sex-specific foraging has been documented in a range of taxa [1]. In seabirds, it is generally considered to arise from competitive exclusion or social dominance of the larger sex, resulting in spatial or temporal segregation of foraging activity and trophic partitioning when the sexes overlap in space and time [2–8]. Yet, sex-specific foraging arises independently of sexual size dimorphism [9–18] suggesting alternative mechanisms, including how males and females cooperate to allocate resources to parental care, in a manner that optimizes reproductive success [19].

Two main hypotheses are proposed to explain the emergence of sex-specific foraging in monomorphic seabirds in relation to parental care. The Reproductive Role Specialization Hypothesis (RRSH) postulates that sex-specific foraging emerges in response to specialized reproductive roles by the sexes [9–14]. For example, Sooty Shearwaters (*Puffinus griseus*) segregate at sea during egg development when females forage in distant productive waters, while males that guard the nest site during this time, forage in close proximity to the colony [14]. The Energetic Constraint Hypothesis (ECH) states that divergent energy requirements by the sexes during one reproductive stage are resolved through sex-specific foraging at another stage [15–18]. For example, females in relatively poor condition after egg-laying [20] may spend more time foraging for themselves, resulting in male-biased offspring provisioning during early chick-rearing [17].

In the present study, we use a combination of geolocation-immersion loggers and stable isotope analyses to investigate sex-specific foraging in a small (1 kg), monomorphic seabird, the Common Murre (*U*. *aalge*), where the male parent alone rears the chick to independence following a brief, but demanding period of bi-parental care at the colony. Although murres exhibit slight sex differences in bill size and body mass [10, 21], we consider them monomorphic since there is no difference in the first principle component of body size [22]. Along with two related members of the tribe Alcini (Thick-billed Murre *U*. *lomvia* and Razorbill *Alca torda*), Common Murres (hereafter murres) exhibit an unusual mode of ‘intermediate’ juvenile development [23]. This developmental mode involves c.a. 21 days of bi-parental care at the colony [24–25], followed by c.a. 2 months of male-only care at sea [26]. Murres are single-prey loaders [25] that experience the highest flight costs of any volant species [27], and operate near their physiological limit during chick provisioning at the colony [27–28]. Consequently, high parental energetic expenditures and limited chick growth potential combine to favor a relatively brief chick-rearing period at the colony [29–30]. Yet, chicks are unable to fly and weigh only 25% of adult weight when they go to sea [25], and are totally dependent on the male parent until they attain nutritional dependence, some 2 months later [26]. After colony departure, flightless male-chick pairs swim quickly away from the colony to offshore nursery areas, and male parents moult their flight feathers [31–33]. After fledging, females continue to attend the colony for c.a. 2 weeks to defend their breeding territory [34], after which they fly offshore to moult their flight feathers and become temporarily flightless [35–36].

There is support for both RRSH [11, 13] and ECH [16, 18, 37] to explain sex-specific foraging by murres but, since most studies are conducted during biparental care, there is relatively less support for RRSH. Paredes et al [12] argue that male Thick-billed Murres are relatively more aggressive than females, and play a greater role in nest defense; brooding the chick overnight when predation risks are highest [10]. As a result, male murres forage primarily during daylight hours when prey is located deeper in the water column, and dive significantly deeper than crepuscular foraging females [11, 13]. However, Elliot et al [18] argue that evidence of site-specific variability in the nest attendance patterns of Thick-billed Murres, with males brooding at night at some colonies but not others; implies that nest attendance patterns are unrelated to nest defense but rather, can be explained by ECH. Specifically, sex differences in nest attendance patterns are driven by diel patterns in the availability of the preferred prey of the sexes, whereby males target risk-averse shallow-water prey (versus risk-prone benthic prey by females), to maintain body condition in preparation for the post-fledging period [18]. Additional support for ECH comes from evidence that flightless male-chick pairs (based on one male only) associate with lower quality foraging areas relative to other independent murres, suggesting a potential constraint on energy intake for male parents during the post-fledging period [37]. Therefore, documented sex differences in the foraging behavior of chick-rearing murres [16, 18] and female-biased chick-provisioning during chick-rearing at the colony [16, 18, 37, 38] may be explained by ECH, whereby male parents forage more for their own benefit during biparental care in anticipation of energetic constraints after colony departure. Further studies are needed, involving larger samples of single-parenting males to draw stronger conclusions bearing on the ECH.

We compare the foraging behavior and trophic position of male and female murres during three successive life-history stages in the annual cycle, each characterized by a different level of parental care by the sexes. These include: 1) bi-parental care (BPC) during late chick-rearing when both males and females contribute to offspring care, 2) male-only parental care (MOC) during the post-fledging period when males are single-parents and females are independent and 2) no parental care (NPC) during winter when both sexes are released from parental care. The overall goal of the study is to investigate differential parental care as an underlying mechanism for sex-specific foraging by monomorphic murres. Specific objectives are to assess how stage-specific differences in parental care influence the foraging behavior of the sexes, including: (1) overlap in core foraging areas, (2) diel foraging patterns, (3) daily foraging effort (i.e. time spent diving); (4) foraging tactics (i.e. dive depth, bottom-time, ascent and descent rates, post-dive interval) and (5) trophic position of the sexes.

If sex differences in the foraging behavior of murres are driven by specialized reproductive roles (RRSH), we expect to find evidence of sex-specific foraging during MOC, when the parental care roles of the sexes diverge the most, versus no sex differences during BPC when parental care is shared and NPC when parental care is absent. If sex-specific foraging is associated with stage-specific energetic constraints (ECH), we expect male parents to invest proportionally more time in self-feeding prior to colony departure with the chick, resulting in sex-specific foraging during BPC.

## Materials and Methods

### Ethics Statement

The study was carried out in strict accordance with ethical guidelines outlined by the Canadian Council on Animal Care, and approved by Memorial University of Newfoundland’s Institutional Animal Care Committee (Permit Numbers: 10-01-WM, 11-01-WM, 12-01-WM, 13-01-WM). Fieldwork was carried our under a Canadian Wildlife Service Migratory Bird Banding permit WAM-10322K. Access to the Funk Island and Witless Bay Islands Provincial Seabird Ecological Reserves was permitted through the Newfoundland and Labrador Parks and Natural Areas Division.

### Study Sites and Logger Fieldwork

Fieldwork with murres was carried out at two Northwest Atlantic colonies: Gull Island in the Witless Bay Ecological Reserve (47°16’N, 52°46W) with c.a. 1632 breeding pairs bp [39] and the Funk Island Ecological Reserve (49°45’N, 53°11’W) with c.a. 470, 000 bp (S. Wilhelm, EC-CWS, pers comm). Lotek LAT 2500 geolocation-immersion loggers (5.9 g with attachment, c.a. 0.7% body mass) were attached to plastic leg bands (Pro-Touch Engraving) with cable ties and placed on the left leg of breeding murres during late chick-rearing (adults with chicks > 10–15 days of age). A Canadian Wildlife Service metal band was attached to the right leg. Fifty-one loggers were deployed from 2009–2013: 15 at Funk Island (2009) and 36 at Gull Island (2010–2013). Instrumented birds were recaptured on the nest in the following breeding season. Upon recapture the logger was removed, birds were weighed with a 1 kg Pesola spring balance and 1 ml of blood was collected from the brachial vein for sex determination [40] and stable isotope analysis [41]. Feathers were also collected on recapture, including one secondary covert and 3–6 breast feathers that provide trophic signals for MOC and NPC, respectively [35, 42]. Throughout deployment and recapture, birds were held in a cloth bag for c.a. 4–6 min with their head covered. Approximately 15 control birds (i.e. no logger attachment) were also captured in each year (n = 61) for comparisons of body mass and trophic position with loggered individuals. This allowed us to assess possible device effects on body condition and foraging behavior.

### Logger processing

#### Activity data

Loggers were programmed to record dry state every 60 s when ambient temperature was < 28°C. To conserve memory, wet state was not logged and a temperature criteria were used to distinguish between dry periods at sea that represent flight, versus dry periods at sea that represent leg-tucking (i.e. when the bird draws its leg and foot into its plumage). This approach assumes that heat transfer from the bird’s body (39.6°C; [43]) during leg-tucking would result in temperature readings approaching 28°C, well above ambient air and water temperatures experienced by North Atlantic murres during the non-breeding period. Patterns in the timing and duration of uninterrupted dry events (> 1 min) were used to determine colony departure (late summer) and arrival (spring) dates, where dry events greater than 360 min (or 6 h) indicated regular colony attendance. In addition, dry periods at night (> 60 min) provided corroborating evidence of colony attendance since murres are known not to fly at night [44]. Sex comparisons of the timing of colony departure and arrival were standardized according to mean dates (± SD), and by year when sample sizes allowed. Limited memory capacity and some mid-year device failures resulted in fewer individuals for spring colony arrival estimates.

#### Positional data

Positional data from Lotek loggers are derived from internal processing algorithms that generate a single daily location, based on measurements of light intensity [45]. Comparisons of Lotek positions with those generated from BAS loggers deployed on murres at the same colonies [46–47] revealed a potential bias, with Lotek positions extending further north than expected in fall, and further south in winter. This bias has been identified by other researchers using Lotek loggers to study the seasonal distribution of North Atlantic murres, and is likely associated with higher light exposure at a given date and latitude than is assumed by the device’s onboard algorithm (M. Frederiksen, pers. comm.). Therefore, using an R script validated by M. Frederiksen, all positions were recalculated by: 1) back-calculating times of sunrise and sunset using the built-in sun angle of -3.44°, and 2) re-estimating latitudes assuming a true sun angle of -5°. Re-calculations were performed using the R package ‘GeoLight’ 2.0 (experimental version provided courtesy of S. Lisovski). Inspection of recalculated positions revealed a significant improvement during fall and winter, aligning with seasonal distributional patterns from previous studies on Newfoundland murres [46–47].

Unfiltered positions were subsequently mapped in ArcMap 10.0 (ESRI, 2010) and inspected visually to remove locations that represented improbable daily movements (i.e. >500 km/day [48]), or were outside the expected non-breeding range of murres [46–47]. Erroneous locations around the vernal (c.a. 9 September—9 October) and spring equinoxes (c.a. 6 March—6 April) were excluded since latitudinal data derived from day length are unreliable during equinox when day lengths are similar around the world [49]. The total number of retained, post-processing positions represented 61% of the original 3452 raw positions.

Fifty percent kernal density contours were used to represent the core foraging areas of males and females during the MOC and NPC [50–51]. BPC was excluded since maximum foraging ranges of breeding birds around the colony (max <80 km; [52]) are less than the 100–200 km mean positional error for geolocation loggers [48]. Kernal home ranges were evaluated for unsmoothed positions using a least squared cross validation method with a 50 km grid size, applying the ‘kernelUD’ function in the ‘adehabitatHR’ package [53] in Cran R (ver. 3.1.2). Percentage overlap of kernal density contours (50%) of females and males within each stage was calculated using the ‘kerneloverlaphr’ function (HR method), that calculates the proportional overlap of one sex relative to the other:

$$HR_{\left(f,m\right)}=A_{\left(f,m\right)}/A_{\left(f\right)}$$

where A_(f,m)_ represents the area of intersection between females and males and A_(f)_ is the home range area of females [53]. Overlap is presented as the mean 50% core area of females and males.

#### Dive data

Loggers were programmed to record pressure every 8 s when submerged below 2 m. Individual dives were analyzed using the dive analysis program, MT-Dive 4.0 (Jensen Software). Estimates of dive depth, bottom duration, ascent and descent rate and post dive interval were derived for all dives (≥3m). Dive bouts were identified according to a bout-ending criterion using an empirical maximum likelihood approach [54] executed in ‘diveMove’ package in R [55]. Post-dive intervals greater than the identified bout-ending criteria for each individual indicated the onset of a new bout. Bottom duration was defined as the time from the first and last instant when vertical velocity (calculated between successive records) fell below 0.5 ms^-1^ [56]. Daily foraging effort of individual murres during each stage was estimated using accumulated dive time per day, or the total time per day.

To investigate sex-specific diurnal patterns in diving behavior, individual dives were assigned to a specific time period (day, twilight, night): day was defined as the period between sunrise and sunset (when the sun angle is above 0°), twilight as the period when the sun is between 0° and -12 (nautical) and night as the period when the sun is below -12°. Sun angle (°) was calculated using astronomical models [57], based on the formula by [58] and executed in Cran R (ver 3.1.2) using an R script validated by P. Regular. Chi-square tests were applied to assess sex differences in diurnal diving activity, expressed as mean number of dives per time period on a daily basis, within each stage.

#### Index of patch quality

To investigate sex differences in prey patch quality within stages, we calculated an index of patch quality (IPQ) for all individual dives (≥3 m) using the formula presented by [37]. IPQ is based on theoretical models of optimal dive behavior for relationships between dive depth, durations and surface pauses [37, 59]. IPQ values increase with increasing bottom time for a given dive depth, based on the assumption that bottom time will be increase only when patch quality is high [59]. To eliminate surface pauses not associated with foraging bouts, we included dives occuring within foraging bouts only, expressed as mean IPQ per dive bout.

### Statistical analysis

The effects of sex, stage, and their interaction on murre foraging effort, diving tactics and IPQ values were examined using generalized linear mixed-effects models (GLMM) with a gamma error distribution. Mixed modeling was used to account for potential pseudo-replication, with individual set as a random effect and an autoregressive variance-covariance matrix (corAR1) to account for the high temporal correlation in the dive data (assessed via autocorrelation function in Cran R). All statistics were run in R (ver 3.1.2) and GLMM models were run using lme4 package [60]. An outcome was considered significant if the confidence interval of a parameter did not include the value of zero effect (using a confidence level of 95%). Model effects and their significance are presented throughout as mean ± SE [95% confidence intervals].

### Stable isotope analysis

Stable isotope analysis of tissues (whole blood and feathers) collected from instrumented and control adult birds at Funk and Gull Islands during 2009–2013 (n = 76 birds) was used to assess trophic position and relative distributions of individual murres. Whole blood that has an isotopic turnover of c.a. 12–15 days [61] provides an isotopic signal for BPC (n = 65, 36 females, 29 males). Flight feathers (secondary covert), grown over an estimated two-month period [35] provided an isotopic signal for MOC (n = 75, 41 females, 34 males). Breast feathers (n = 78, 43 females, 35 males) that provide a late winter, pre-breeding isotopic signal were used for NPC [42]. No appropriate tissue that reflects trophic information during early winter can be sampled from a live murre, therefore late-winter values were assumed to represent an equivalent signal for NPC.

Following Cherel et al [62], feathers were cleaned of surface contaminants using a 2:1 ratio solution of chloroform:methanol. Feathers were air-dried under a fume hood and cut into fragments, avoiding the quill and shaft. Blood samples that were preserved in 95% methanol were oven-dried to a constant mass at 60°C. Dried samples were then coarsely ground, and lipids extracted using a 2:1 chloroform:methanol solution. A 1 mg subsample of feather and blood samples were weighed and placed in a tin cup. Instruments were cleaned with acetone between samples to prevent cross-contamination. Isotope values were determined by the Stable Isotope Facility, University of California, Davis. Results are reported in delta notation (δ) in parts per thousand (‰) relative to air (δ^15^N) and PeeDee Belemnite (δ^13^C). Replicate measurement of laboratory standards (2 standards for every 12 unknowns) indicated measurement errors of ~0.16 and 0.03‰ for nitrogen and carbon, respectively.

Two diet-feather fractionation values were used to convert feather isotopic values to trophic values: 3.7‰ (δ^15^N) and 1.9‰ (δ^13^C) for secondary feathers and 3.6‰ (δ^15^N) and 2.5‰ (δ^13^C) for breast feathers [63]. Average diet-blood fractionation of 2.63‰ (δ^15^N) and 0.37‰ (δ^13^C) were used for whole blood [64]. All fractionation factors were subtracted from raw isotopic values prior to analysis.

Analysis of variance (ANOVA) was used to examine sex-specific differences in δ^15^N and δ^13^C values of murres within each stage. Repeated measures ANOVA were applied to examine differences in the δ^15^N and δ^13^C values for each sex across stages, using a linear mixed-effects model executed in the R package lme4 [60]. Post-hoc Tukey’s multiple comparison test for unequal sample sizes were applied to statistically significant outcomes. Statistical significance was defined as *p* < 0.05.

### Delineation of seasonal life history stages

Behavioral information derived from data loggers was used to define the three life history stages in the annual cycle for all individuals. BPC was defined as the period from logger deployment to colony departure (males) and for females, BPC was defined as the period from logger deployment to one week before colony departure [34]. MOC was defined as the period from colony departure to offspring independence for males and females, where offspring independence was estimated based on the daily dive rates of single-parenting males (self and chick-provisioning), that is expected to be higher than for independent females [37]. The no-parental care stage (NPC) was defined as 1 November to 31 January (or the end date of the pressure log). S1 Table provides detailed information on the range of sampling dates according to each stage for all individuals (dive data only).

## Results

### Device effects and data availability

Following capture and deployment of devices, birds returned consistently to their breeding site either immediately or soon after release (within 30 min), suggesting that levels of disturbance due to capture and handling were surmountable. Mass at recapture did not differ between instrumented (982.6 g ± 54.8 SD, n = 29) and control (977.1 g ± 76.3 SD, n = 61) birds (t_76_ = 0.4, *p* = 0.7). There was no significant difference (F_1,255_ = 0.4, *p* = 0.5) in the mean δ^15^N values of loggered and control birds during MOC (F_1,75_ = 1.9, *p* = 0.2) or NPC (F_1,78_ = 1.8, *p* = 0.2). These results suggest that the body condition and foraging behavior were not negatively affected by carrying devices over the year.

Of 51 loggers deployed on breeding murres, 29 were retrieved (3 of 15 at Funk I and 26 of 36 at Gull I). Four of these devices failed, resulting in a final sample size of 25 individuals consisting of 15 females, 9 males and one individual of unknown sex that was excluded from analyses (Table 1). In addition, the chick of one male parent in 2011 was confirmed lost when both parents were observed on the breeding site after fledging. This unsuccessful male was treated separately from other males during MOC (Table 1). Inconsistency in logger performance resulted in a variable sample distribution across data types and life-history stages (Table 1). Eighteen of 25 loggers did not successfully record all three data logs, resulting in partial behavioral information for some individuals (Table 1). In addition, dive information was unavailable for BPC and MOC for 4 individuals in 2013 (3 F, 1 M), since loggers were programmed to start recording pressure on 1 November (versus at deployment) to capture foraging behavior through winter and spring.

**Table 1:** Deployments (Out), retrievals (In) and data outcomes by site, year, sex and log type. Numbers refer to individuals. Letter superscripts explain discrepancies in sample size.

Year	Out	In	With Data	Wet/dry			Light			Light		
				#	F	M	#	F	M	#	F	M
**2009**	15	3^A^	3^B^	2 (2^C^)	2 (2^C^)	0	3	2	1	3	2	1
**2010**	8	7	7	7	4	3(1^UM^)	7	4	3 (1^UM^)	7	4	3 (1^UM^)
**2011**	11x	7	4^B^	3	2	1	4	2	2	3	2	1
**2012**	6	3	2^B^	2	1	1	1	1	0	2	1	1
**2013**	11	9	9^B^	9 (1^U^)	6	2	8 (1^U^)	5	3	8 (1^U^)	5 (3^W^)	2 (1^W^)
**Totals**	**51**	**29**	**25**	**23 (1** ^U^ **)**	**15**	**7**	**23 (1** ^U^ **)**	**14**	**9**	**23 (1** ^U^ **)**	**14**	**8**

^**A**^ Arctic fox disturbance (details in [65])

^B^ partial (i.e. < 3 logs)

^C^ <10 days

^UM^ unsuccessful male (i.e. not accompanied by chick at sea)

^U^ unknown sex

^W^ dive data recorded after 1-Nov.

Overall a combined total of 117,052 dives (≥3m) were recorded over three stages, with an average of 7580.7 ± 2661.6 dives from 14 females and 8865.3 ± 2939.2 dives from 8 males. We found no significant sex difference in the overall number of dives (independent samples t test: t_16_ = 0.8, *p* = 0.3). A total of 2634 bird days were sampled, with a mean sampling period of 121 days for females and 117 days for males (*p* = 0.80). There was no sex difference in the number of bird days samples within stages, but across stages the sampling distribution was shorter during BPC for both sexes, relative to MOC and NPC (S1 Table).

### Timing of colony departure and arrival

Independent females departed the colony later than males in all years, with an average difference of 7 days (Table 2). The male of a breeding pair (2012) departed the colony on 25 July, 12 days earlier than the female partner (06 Aug; Table 2). Murres returned consistently to the colony in early-mid May with earlier arrival dates by males in all years by an average difference of 6 days (Table 2).

**Table 2:** Summary of colony departure and arrival dates of individually tracked murres (wet-dry log). Values are median dates (± SD days) summarized by sex (and year where sample size allows).

Year	Colony Departure Median Date		Colony Arrival Median Date	
	F (n = 15)	M (n = 7)	F (n = 8)	M (n = 4)
2009	22 Aug ± 0 d (2)	16 Aug (1)	NA	NA
2010	21 Aug ± 4.6 d (4)	17 Aug ± 10 d (2)	20 May ± 4 d (2)	15 May ± 1 d (2)
2011	11 Aug ± 2 d (2)	31 Jul (1)	24 May (1)	06 May (1)
2012^P^	06 Aug (1)	25 Jul (1)	17 May (1)	NA
2013	07 Aug ± 2.5 d (6)	30 Jul ± 8 d (2)	08 May ± 7 d (4)	03 May (1)
Overall	10 Aug ± 7.5 d	3 Aug ± 11.1 d	16 May ± 7.1 d	10 May ± 5.5 d

^P^ breeding pair sampled in 2013.

### Sex-specific overlap in core foraging habitat

**Fig 1:**
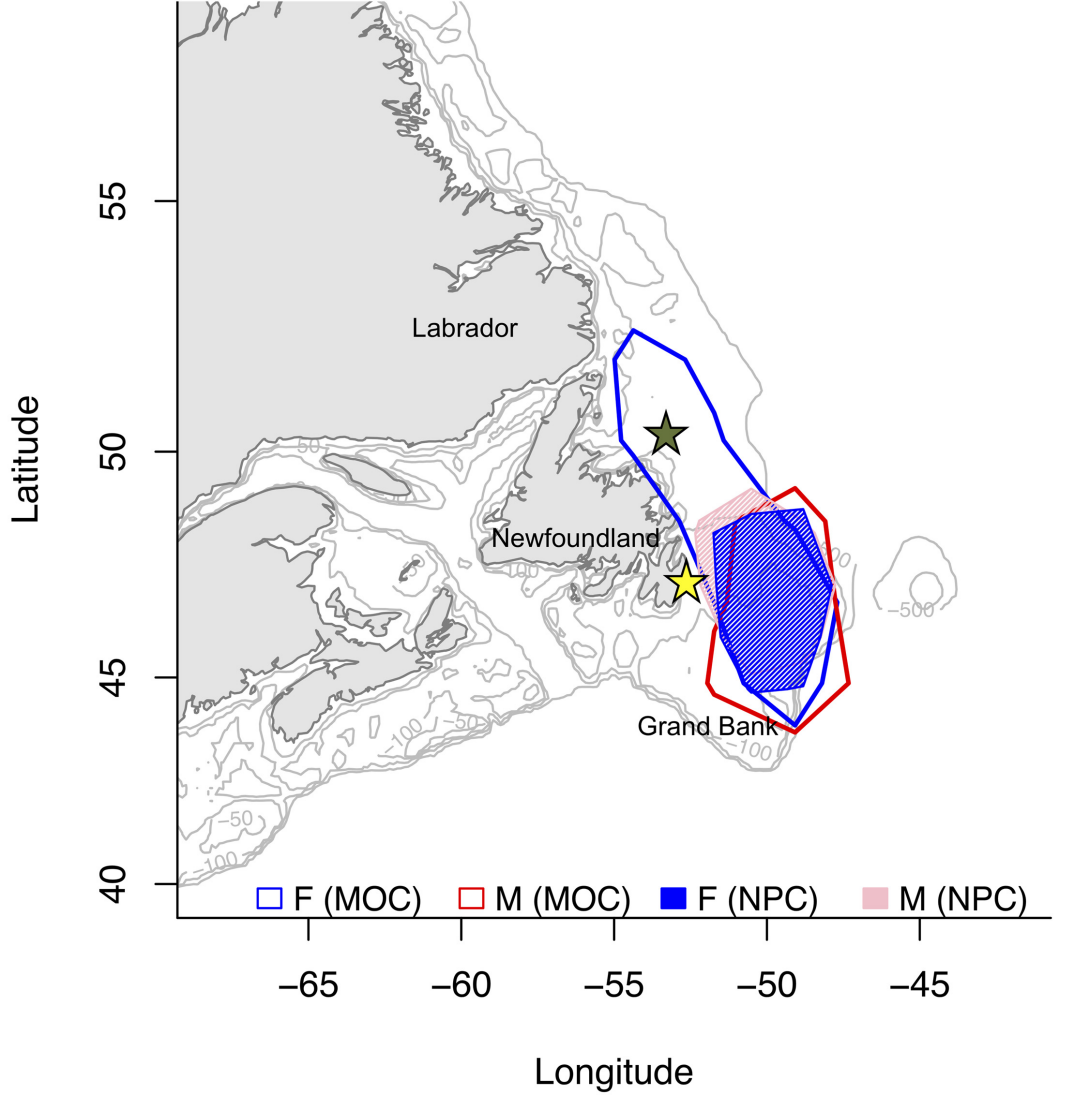
Core foraging areas (50% kernal contours) of female (blue) and male (red) murres during MOC (14 F, 8 M) and NPC (12 F, 9 M). Colony locations are indicated by a yellow (Gull I) and green star (Funk I). Depths between isobaths are 100 m. Bathymetry data were obtained online from the GEBCO Digital Atlas (GEBCO one-minute grid, ver. 2, www.gebco.net).

There was only partial overlap in the core foraging areas (represented by 50% kernal density contours) of female and male murres during MOC (39.3%), overlapping on the southern Grand Bank of Newfoundland (Fig 1). Independent females utilized a larger area relative to single-parenting males (Table 3), extending into near shore and shelf waters on the Newfoundland-Labrador shelf (Fig 1). During NPC, the core foraging areas of females and males overlapped extensively (85.5%; Table 3) on the southern Grand Bank (Fig 1).

**Table 3:** Seasonal kernel home range areas (KHR; km^2^) and percentage overlap of core foraging areas (50% kernal distribution) of female and male murres during MOC and NPC.

Stage	N		50% KHR (km^2^)		Mean Percentage Overlap KHR (50%)
	F	M	F	M	F,M
MOC	14	8	476,664	313,856	39.3%
NPC	12	9	222,747	222,404	85.5%

### Index of Patch Quality

**Fig 2:**
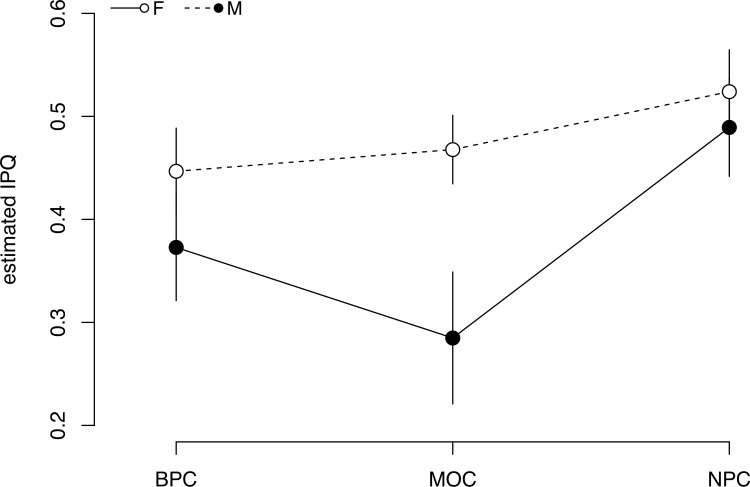
Mean (SE) index of patch quality (IPQ) for all within bout dives (≥3 m) by female and male murres across successive stages of the annual cycle. Values are GLMM model fits ± confidence intervals. Sample sizes provided in S1 Table.

Sex had a significant effect on IPQ during MOC only (Fig 2), with lower IPQ values for males (0.28 ± 0.03, [95% CI: 0.22–0.34]) than females (0.46 ± 0.02, [95% CI: 0.44–0.50]). IPQ values of females were higher than males during BPC (0.44 ± 0.02 and 0.37 ± 0.02 for females and males respectively) and NPC (0.52 ± 0.02 and .49 ± 0.02 for females and males respectively), but the difference was not significant in either stage (Fig 2).

### Sex-specific diurnal foraging

**Fig 3:**
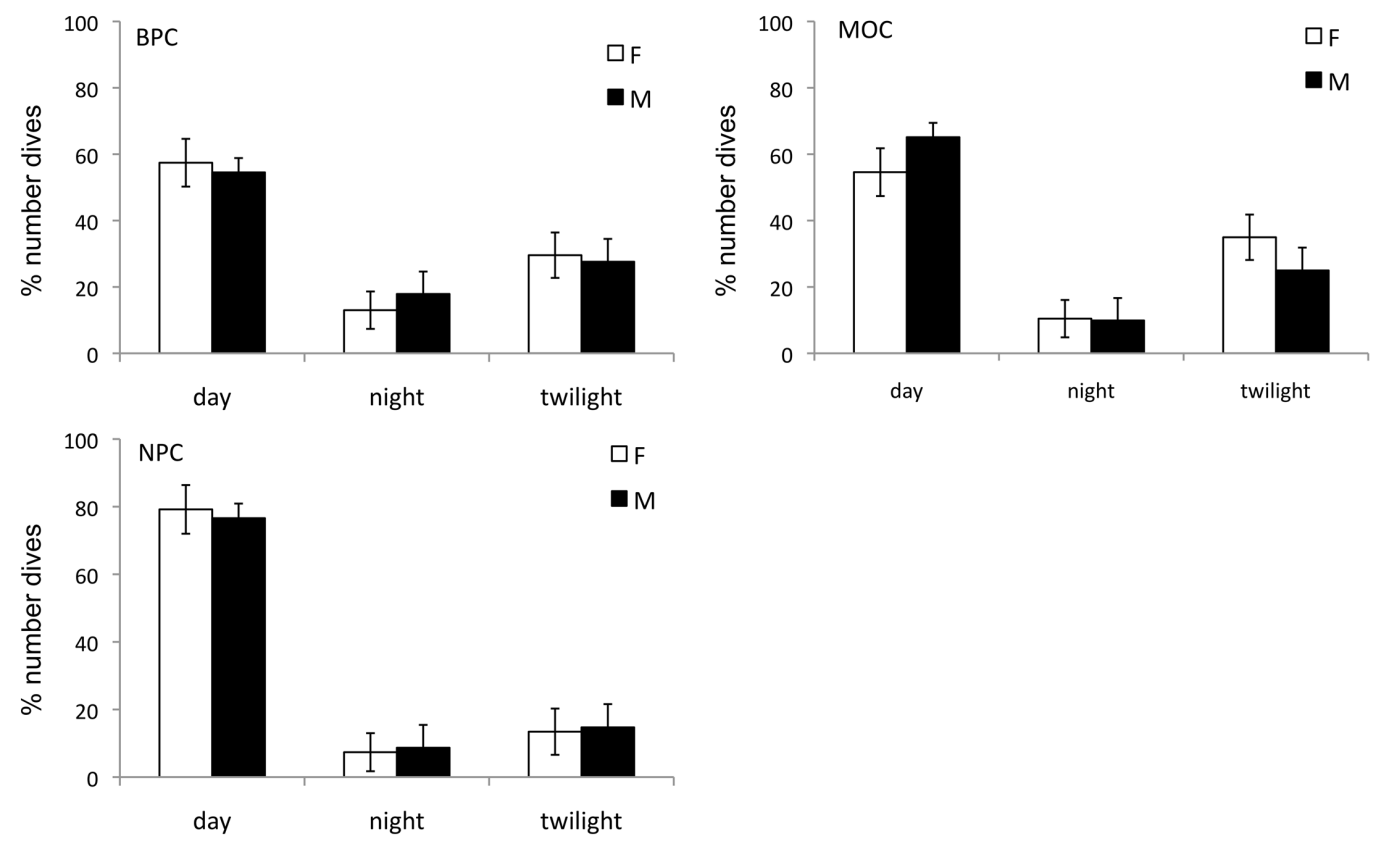
Mean percent daily number of dives by female and male murres according to light phase during BPC (top left), MOC (top right) and NPC (bottom left). Values are mean ± SE percent total number of dives day^-1^ across individuals (χ^2^ tests: *p* < 0005). Sample sizes provided in S1 Table.

Sex differences in the daily timing of foraging were found during MOC only (χ^2^ = 6.7, *p* = 0.03), when males dove more than females during daylight (65.1 ± 2.9% and 54.6 ± 3.8%, females and males respectively) and less than females during twilight (25.0 ± 4.0% and 35.0 ± 4.1%, %, females and males respectively; Fig 3). Overall, murres dove most frequently during daylight hours (Fig 3), and the frequency of daylight dives increased during NPC, accounting for 79.2 ± 3.3% of dives by females and 76.6 ± 4.6% by males. Night diving occurred during all stages with no significant effect of sex, but was most frequent during BPC for females (13 ± 5.6%) and males (17.9 ± 6.8%; Fig 3).

### Sex-specific foraging effort

**Fig 4:**
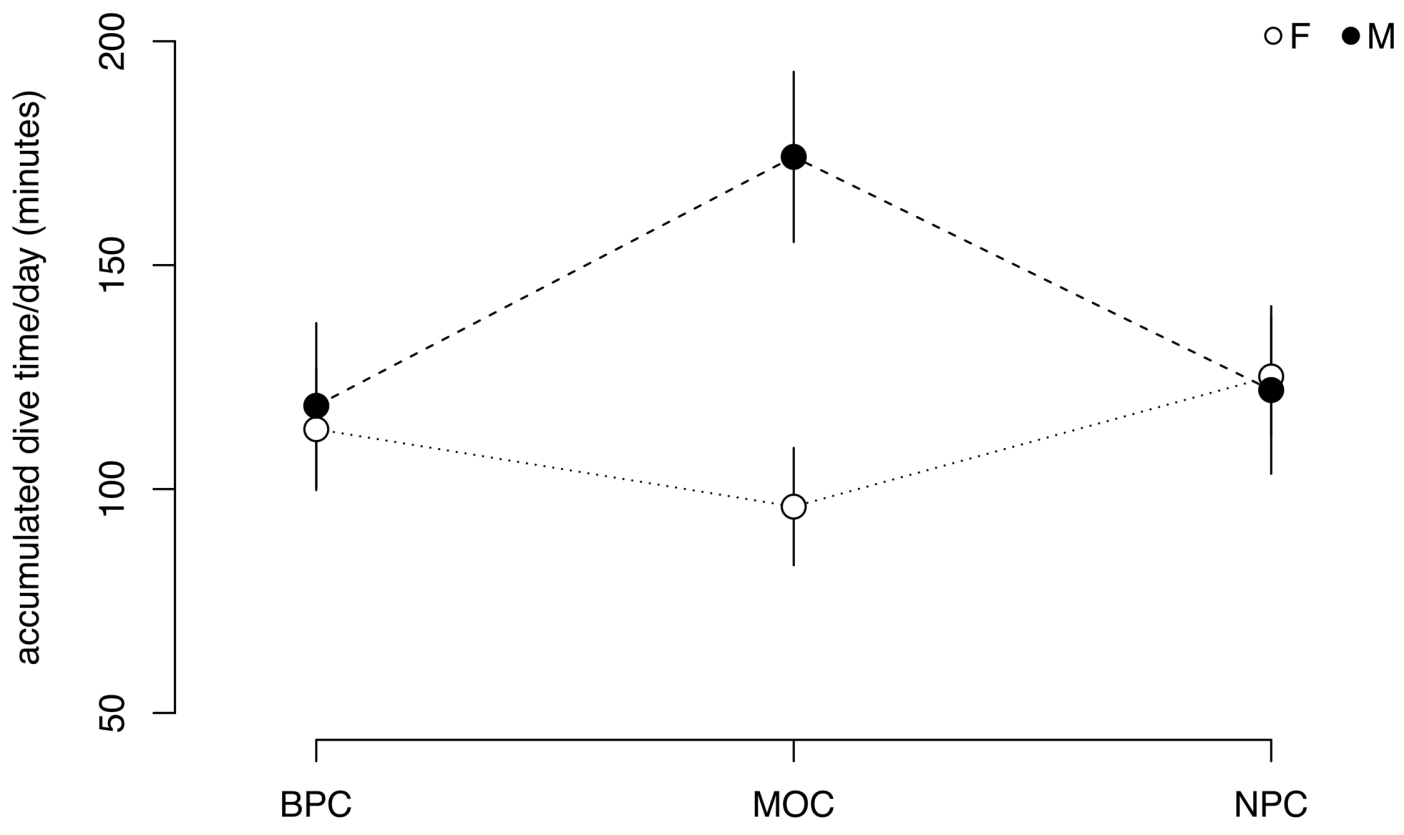
Mean accumulated daily dive time of female and male murres across successive stages of the annual cycle. Values are GLMM model fits ± confidence intervals. Sample sizes provided in S1 Table.

Sex had a significant effect on foraging effort (i.e. accumulated daily dive time) during MOC only (Fig 4), when males spent almost twice as much time diving per day (174.2 ± 9.7 min day^-1^, [95% CI: 155.2–193.2 min day^-1^]) than independent females (96.1 ± 6.9 min day^-1^, [95% CI: 82.6–109.6 min day^-1^]). There was no effect of sex on the time spent foraging during BPC or NPC (Fig 4). Daily time spent foraging by females was similar across stages (Fig 4), but males exhibited a significant increase in time spent foraging from BPC (118.6 ± 9.4 min day^-1^, [95% CI: 100.2–137.1 min day^-1^]) to MOC (174.2 ± 9.7 min day^-1^), followed by a subsequent decrease during NPC (122.1 ± 9.5 min day^-1^, [95% CI: 103.4–140.8 min day^-1^]). Daily foraging effort was slightly lower during BPC (113.4 ± 6.9 min day^-1^ and 118.6 ± 9.4 min day^-1^, females and males respectively) than NPC (125.2 ± 6.8 min day^-1^ and 122.1 ± 9.5 min day^-1^ respectively), despite significantly shorter days in winter (Fig 4).

### Sex-specific diving behavior

There was a significant effect of sex on dive depth during MOC only, with males (48.6 ± 0.8 m) diving deeper than females (35.5 ± 0.7 m; Table 4). During BPC, mean dive depth of males (58.6 ± 5.8 m) was greater than females’ (50.0 ± 4.5 m), though the difference was not statistically significant. Mean dive depths of females (51.6 ± 0.8 m) and males (50.2 ± 1.2 m) were strikingly similar during NPC (Table 4). Mean dive depth of females decreased significantly from BPC (50.0 ± 4.5 m) to MOC (35.5 ± 0.7 m), and increased again in NPC (51.6 ± 0.8 m). Males dove significantly deeper during BPC (58.6 ± 5.8 m) relative to MOC (48.6 ± 0.8 m) and NPC (50.2 ± 1.2 m). Dive depth and dive duration were highly correlated (r^2^ = 0.92, *p* < 0.0001), so only information on dive depth is presented here.

**Table 4:** Dive characteristics (using the mean value per bout) of female and male murres during three stages of the annual cycle. Values are GLMM model fits (mean ± SE) and 95% CI (in brackets) by sex according to stage. Bold indicates significant within stage sex differences, with the highest value in bold.

Diving parameters	BPC (n = 18)		MOC (n = 17)		NPC (n = 19)	
	F (11)	M (7)	F (11)	M (6)^3^	F (12)	M (7)
depth (m)	50.0 ± 4.5 (41.2–58.7)	58.6 ± 5.8 (47.3–69.9)	35.5 ± 0.5 (33.6–37.4)	**48.6 ± 0.8 (47.1–50.1)**	51.6 ± 0.8 (50 .0–53.1)	50.2 ± 1.2 (47.9–52.4)
bottom time^1^ (s)	**31.9 ± 0.1 (31.8–32.1**)	27.0 ± 0.1 (26.8–27.3)	**28.8 ± 0.2 (28.3–29.2)**	24.9 ± 0.5 (23.9–26.0)	**45.6 ± 0.3 (45.1–46.2)**	43.2 ± 0.4 (42.4–44.0)
descent rate (ms^-1^)	1.2 ± 0 (1.2–1.3)	1.3 ± 0 (1.3–1.4)	1.0 ± 0 (1.0–1.1)	**1.2 ± 0 (1.2–1.3)**	1.2 ± 0 (1.1–1.2)	1.2 ± 0 (1.1–1.3)
ascent rate (ms^-1^)	1.3 ± 0 (1.3–1.4)	1.3 ± 0 (1.3–1.4)	1.1 ± 0 (1.1–1.2)	1.2 ± 0 (1.2–1.3)	1.3 ± 0 (1.2–1.3)	1.3 ± 0 (1.2–1.3)
post-dive interval^2^ (s)	151.6 ± 19.8 (111.8–191.4)	160.6 ± 27.4 (105.5–215.7)	110.2 ± 18.7 (72.4–148)	144.8 ± 30.6 (82.3–205.8)	174.4 ± 20.7 (132.6–216.2)	191.5 ± 32.2 (126.2–256.5)

^1^excludes bottom time = 0.

^2^dives with post dive intervals > 25 minutes.

^3^ excludes 1 male whose chick was lost.

There was a significant effect of sex on mean bottom duration during all stages, with females spending more time on the bottom phase of dives relative to males (Table 4). Mean dive descent rate of males was faster than females during all stages, but the difference was only statistically significant during MOC (Table 4). There was no effect of sex on dive ascent rates or post dive interval during any stage (Table 4). Despite longer post-dive intervals during NPC for females (174.4 ± 20.7 sec) and males (191.5 ± 32.2 sec), there was no significant difference across stage for either sex (Table 4).

### Sex-specific trophic position

**Fig 5:**
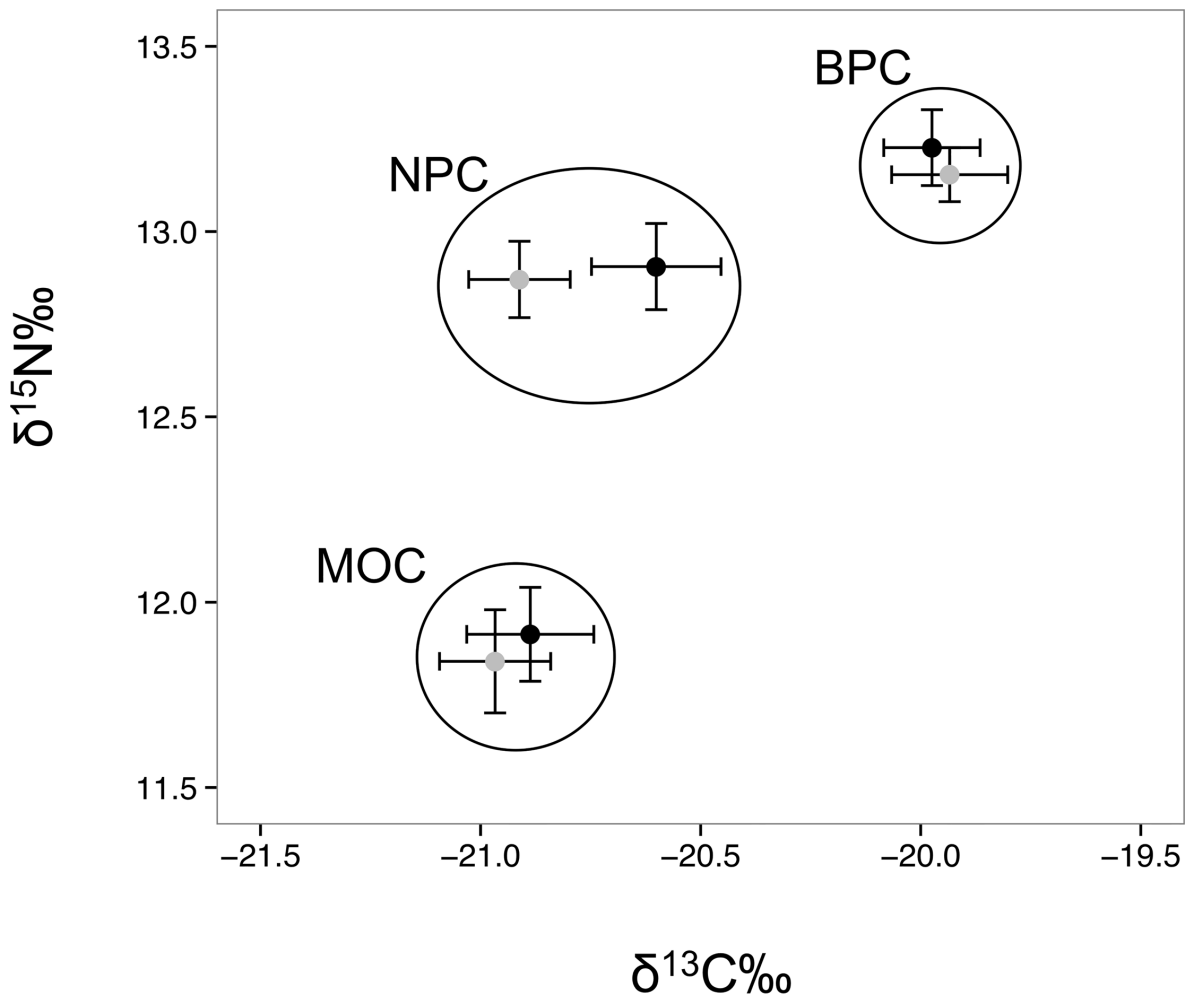
Stable isotope values (fractionation adjusted) of murres according to sex and stage. δ^15^N‰ and δ^13^C‰ (mean ± SE) values of blood (BPC; n = 65), secondary covert (MOC; n = 75) and breast (NPC; n = 78) feathers of female (grey) and male (black) murres.

There was no effect of sex on mean δ^15^N isotopic values (fractionation adjusted; mean ± SD) derived from blood (representing BPC; F_1,63_ = 0.4, *p* = 0.6), secondary coverts (representing MOC: F_1,73_ = 0.1, *p* = 0.7) or breast feathers (representing NPC: F_1,75_ = 0.4, *p* = 0.6; Fig 5). Mean δ^15^N values in secondary coverts of females (+11.8 ± 0.9‰) and males (+11.9 ± 0.7‰) were significantly lower than those of blood (+13.2 ± 0.4‰ and +13.2 ± 0.6‰ and for females and males respectively) and breast feathers (+12.9 ± 0.7‰ for females and males; Fig 5).

Mean (± SD) δ^13^C isotopic values (fractionation adjusted) in blood and secondary feathers of females and males did not differ (Fig 5), but δ^13^C in male breast feathers (NPC: -20.5 ± 0.8‰) was significantly higher (F_1,75_ = 4.3, *p* = 0.04) than in female breast feathers (-20.9 ± 0.8‰). For females, overlapping δ^13^C values in flight (-21.0 ± 0.8‰) and breast feathers (-20.9 ± 0.8‰) were significantly lower relative to blood (-19.9 ± 0.8; F_2,79_ = 41.5, *p* <0.001). There was no significant overlap in male blood, flight or breast feathers (F_2,60_ = 52.5, *p* <0.001).

## Discussion

We investigated differential parental care as a mechanism for sex-specific foraging in monomorphic Common Murres, evaluating two hypotheses: the reproductive role specialization hypothesis (RRSH) and the energetic constraint hypothesis (ECH). Our results demonstrate sex-specific foraging during the stage with the greatest divergence in parental care roles (MOC) versus a convergence in foraging behavior during biparental care (BPC) and winter when parental care is absent (NPC). These findings support RRSH as a mechanism for sex-specific foraging in monomorphic murres.

We also demonstrate a potential energetic constraint for single-parenting males, emerging directly from the need to remain with and provision a flightless, growing chick. Yet, contrary to the predictions of ECH; there was no evidence that male parents spent more time foraging for their own benefit before colony departure in anticipation of demanding conditions after fledging. These findings are not in agreement with previous studies that demonstrate male-biased self-feeding [16, 18, 37], female-biased offspring provisioning [16, 18, 37, 38] and differential prey specialization by the sexes [18] during the late chick-rearing period (>15 days). We hypothesize that discrepancies in our results reflect context-specific foraging conditions that constrain male parental ability to allocate additional resources for their own benefit during BPC when food is relatively scarce.

Chick-rearing murres at Newfoundland colonies are known to specialize on capelin (*Mallotus villosus)* [28, 33, 66–68], and the mean δ^15^N values of male (+13.23‰) and female (+13.15‰) murres during BPC likely correspond to a common diet of capelin [69]. Furthermore, samples of chick feeds collected concurrently at the colony confirm that capelin were the dominant prey in chick diets during the study [28]. Capelin is a lipid-rich, schooling fish that forms predictable spawning aggregations during the summer in Newfoundland, typically overlapping with the peak chick-rearing period of murres and other seabirds [28, 70–71]. It is well established that the parental foraging behaviors and reproductive success of chick-rearing murres throughout the Northwest Atlantic are regulated by the timing and abundance of capelin [70, 72–74]. Murres exhibit resilience to temporary declines in capelin availability, primarily mediated through flexible adjustments in their time budgets and foraging behaviors [72, 75]. In particular, the co-attendance time of murre parents represents a highly flexible aspect of their daily time budget that allows them to buffer reductions in prey availability by allocating more time to finding food during periods of scarcity [75]. Therefore, evidence of significant decreases in the co-attendance times of parental murres at Gull Island in recent years (ranging 1.4 hr/day – 4.9 hr/day during 2007–2010; [28]), relative to the 1980s (mean 3.7 hr/day’ [72, 74]) suggests that chick-rearing murres may be working harder during peak chick demand than in previous decades.

Observed decadal differences in the co-attendance time of murres could potentially reflect an increase in the frequency of prey mismatch years, whereby the timing of the inshore arrival of spawning capelin does not overlap with the peak hatching dates of murres. In fact, during four of the past eight years (2007–2014), there has been a mismatch in the timing of the inshore arrival of capelin and peak chick-rearing at Gull Island ([28, 75, 76], A. Storey, pers. comm.). Furthermore, estimates of the daily energy expenditures (DEE) of murres at Gull Island indicate that even in prey match years the average DEE of parental murres, estimated at 1969.9 KJ/day [28], approaches the theoretical upper limit to sustainable energy expenditure (7 X BMR; [28, 77]). Therefore we hypothesize that when food is relatively scarce, male parents may be unable to gather sufficient resources during peak demand to successfully provision their offspring and accumulate reserves for their own benefit. Consequently, male parents could enter the post-fledging stage with an energy deficit during poor food years, and incur potential fitness consequences.

Yet, some limitations in our data could impede our ability to detect differences in the foraging behavior of the sexes during BPC. For example, while we found no sex difference in the time spent foraging during BPC, it is not known whether males and females allocated their foraging time at sea differently between self-feeding and finding food for their chick. In addition, low spatial resolution during BPC did not allow us to assess whether males and females segregated at sea, or whether males engaged in longer, self-feeding foraging trips as shown previously for murres [16]. However, since murre parents at Newfoundland colonies consume the same prey they fed to their chick, involving an average of c.a. 3 chick feeds per day [28, 78]; it is unlikely that the time allocated to finding food for the chick constituted a significant proportion of the total time spent foraging at sea. As well, preliminary information on the foraging ranges of female (n = 4) and male (n = 5) chick-rearing murres at Funk Island (2014), derived from GPS tracking data (PMR and WAM unpubl. data), indicate no sex differences in foraging distributions or maximum foraging ranges, suggesting that males and females do not segregate at sea during BPC.

**Fig 6:**
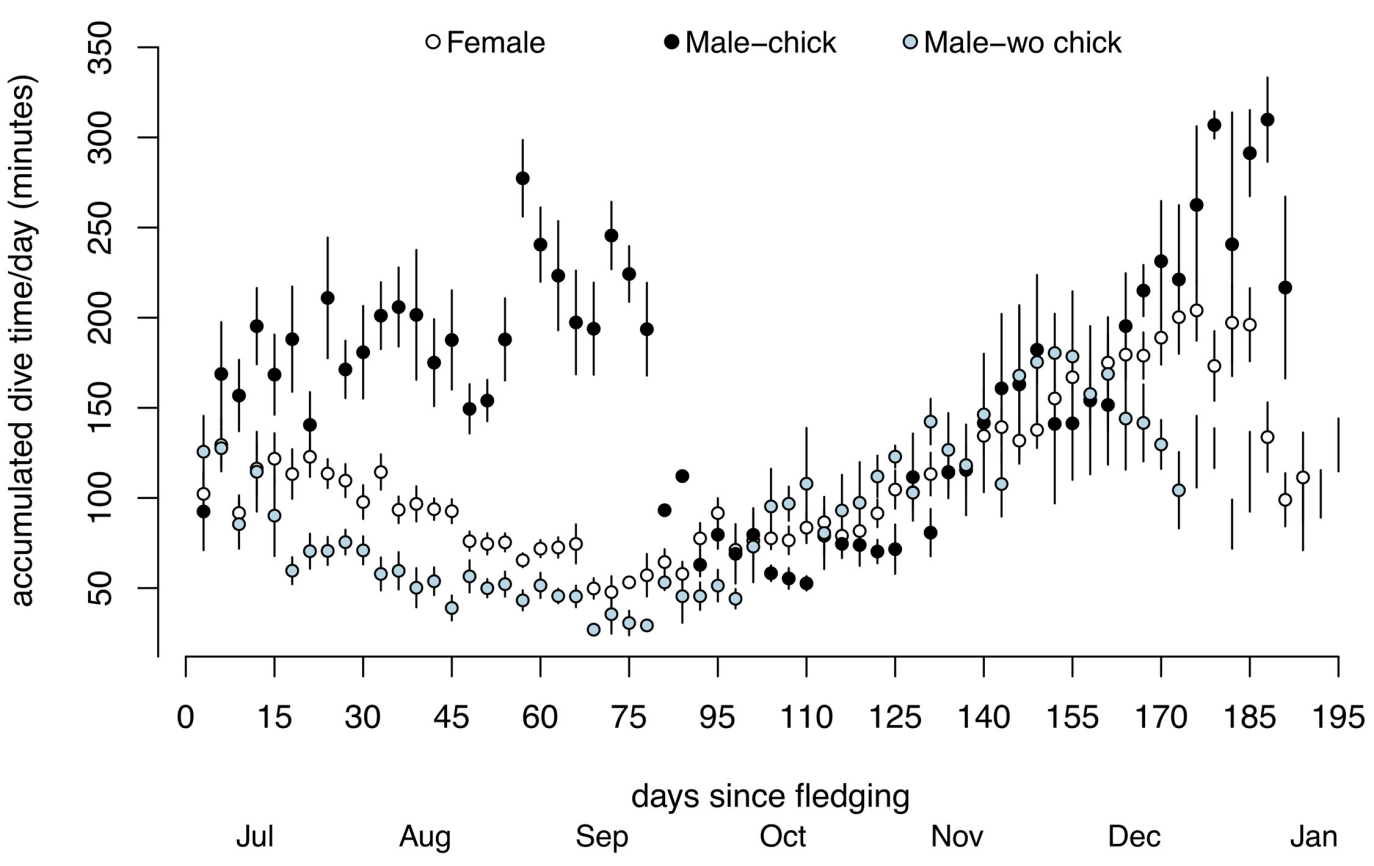
Mean time spent foraging (accumulated dive time; mean ± se) of independent females (n = 11; white circle), single-parenting males (n = 6; black circle) and one unsuccessful male (blue circle) from logger deployment to 31 Jan, showing one data point every third day.

During MOC, single-parenting males spent almost twice as much time diving per day than independent females. Similar behavior was found for Thick-billed murres in the Canadian Arctic [37], with one single-parenting male performing more than twice as many dives per day than other independent murres. The significant increase in the foraging effort of single-parenting males after colony departure likely reflects the nutritional requirements of their growing chick, and provides a useful indicator of the duration of offspring nutritional dependence. Fig 6 shows that single-parenting males spent significantly more time diving per day compared to females, over an average period of 62.8 ± 8.9 days after colony departure (S1 Table). This was followed by a convergence in the daily time spent foraging by the sexes, which we suggest corresponds to the onset of offspring nutritional independence. In support, one male parent whose chick was lost after fledging did not show an increase in foraging effort following colony departure but rather behaved similarly to independent females (Fig 6). The observed range in the duration of offspring nutritional dependence (range 51–75 days; S1 Table) suggests that the time to offspring independence is variable. Whether this is due to inter-annual variability in environmental conditions or individual quality is difficult to determine here, given the small number of individuals sampled over multiple years. Further research is needed to elucidate the circumstances that influence the duration of chick dependence in alcids with intermediate chick development.

**Fig 7:**
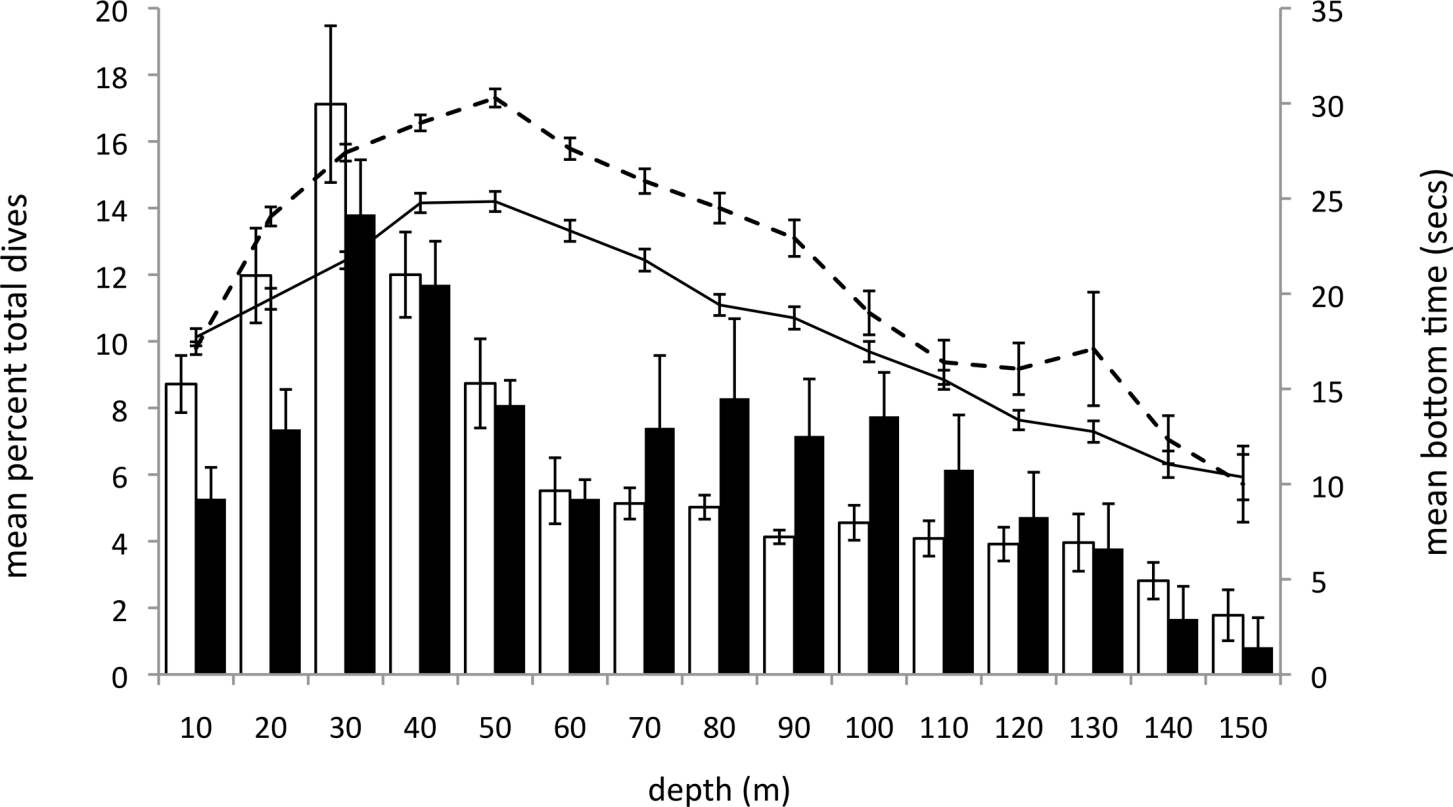
Mean percentage daily number of dives (bars) and mean bottom duration (lines) by 10 m intervals for female (white bars, solid line) and male (black bars, dashed line) murres during MOC.

The mean δ^15^N values of males and females during MOC likely corresponds to a common diet of crustaceans (e.g. shrimp, amphipods, euphausiids) or similar trophic level prey [33]. This indicates a decline in the trophic position of adult murres from BPC (capelin) to MOC (crustaceans), but whether a similar dietary shift occurred for the chicks is unknown. Yet, given the high caloric requirements of a rapidly growing chick (i.e. 13–15 g day^-1^ [79]) in a cold ocean, and the known limits imposed on chick development and body mass deposition by low-lipid diets [80], we expect that growing chicks have nutritional requirements that differ from adults. Furthermore, crustaceans have lower caloric content than fish [81] and are therefore likely unsuitable for rapidly growing chicks. A higher frequency of deep dives by single-parenting males compared to females (Fig 7), despite strong overlap in adult diet, suggests that single-parenting males may dive deep to access alternative prey for their chick. Comparative analysis of δ^15^N values derived from the primary flight feathers of juveniles grown during the post-fledging period ([35]; shot during winter murre hunt in Newfoundland) with post-breeding adult flight feathers could provide insight into the food requirements of growing chicks, and inform our understanding of this critical stage of juvenile development.

Females also dove deep (i.e. > 50 m) during MOC, but much less frequently than males (Fig 7). Rather, females dove more frequently in shallow waters (<30 m) where they spent comparitively more time on the bottom phase of dives (Fig 7). Since the bottom phase of the dive represents the feeding portion of the dive for murres [59], evidence of significantly shorter bottom times by females in relatively shallow water suggests that independent females may have higher dive efficiency and energy intake rates relative to single-parenting males. Males may spend less time on the bottom phase of dives if prey is scarce or patchy. Relatively lower IPQ values for single-parenting males support this, and suggests that the dive efficiency and energy uptake of male parents may be constrained by poor foraging conditions during MOC. Because males are rendered flightless immediately upon departure with their flightless chick, they have limited opportunites to locate high quality foraging areas. Even if they have prior knowledge of ocean nursery areas (using memory; [82]), they must still initiate long risky swimming migrations to offshore areas [31], during which opportunities to locate quality foraging areas may be limited. In contrast, females that spend brief periods at the colony after the male-chick pairs depart (7 days in this study), and can fly during the first days at sea, may use this time to prospect high quality foraging areas for the upcoming period of moult-induced flightlessness.

Alternatively, shorter bottom durations by single-parenting males could also reflect parental vigilance, whereby single-parenting males reduce the time spent on the bottom phase of dives to minimize the total time the chick is left unattended at the surface. This behavior would persumably decrease in importance over time however, as chicks grow larger and become increasingly less vulnerable to avian predators or harassment by other murres. Accordingly, we would expect to observe an increase in bottom duration over time, but this is not supported by our data that shows no decreasing trend in mean bottom duration over time (CMB unpubl. data).

Overall, our results suggest that single-parenting males may experience a significant energetic constraint during MOC. However, the corresponding temporary elimination of flight costs imposed by behavioral flightlessness and prebasic moult may allow males to overcome the constraints associated with offspring provisioning at sea. In support, estimates of the daily energy expenditures of Thick-billed murres indicate low expenditures during prebasic moult, relative to colony-based chick-rearing and winter [37, 83]. Yet, if male parents are unable to allocate additional reserves to self-maintenance before they depart the colony, prolonged energy deficits over the post-fledging period could potentially incur fitness consequences.

During NPC, there was no difference in the foraging behavior of male and female murres. The convergence in foraging behavior during a period with no parental care by either sex suggests that sex differences parental care is a major driver of sex-specific foraging by murres. However, similar foraging behavior by the sexes during winter could also reflect the minimum level of effort required to survive under conditions of increasingly limited food and light availability during North Atlantic winters. Despite significantly shorter days in winter, there was no difference in the time spent foraging during BPC and NPC. This suggests that murres may be forced to maximize foraging time during short days in winter to achieve their daily energy requirements, are high in winter due to thermoregulatory costs [83]. Therefore, intense foraging during short, cold days may provide murres with the needed energy to overcome periods of fasting during long winter nights, and severe storms that temporarily impede foraging ability [84]. The winter foraging ecology of murres is poorly known, and further studies investigating behavioral strategies in response to harsh and limiting winter conditions are warranted.

## Conclusions

Our study demonstrates sex-specific foraging behavior in monomorphic Common Murres during a 2-month period of post-fledging, male-only parental care (MOC) when single-parenting males work hard to provision a growing chick under marginal foraging conditions. Despite this, males did not spend more time foraging for themselves before colony departure in anticipation of difficult conditions during the post-fledging period. If chick-rearing murres face unsustainable energy expenditures during prey-mismatch years, as has been shown for Newfoundland murres that rely on capelin [28]; male parents may be unable to gather sufficient resources during peak demand to successfully provision offspring, and prioritize their own condition. If so, male parents could theoretically enter the post-breeding stage at an energy deficit during poor food years, and incur fitness consequences. Further research is needed to characterize the energy constraints associated with all aspects of parental care in murres to improve understanding of sex-specific responses to seasonally changing parental and environmental constraints.

## Supporting Information

S1 Table Summary information for dive data according to individual and sex, showing the start and end dates for each life history stage. Letter subscripts provide additional information. Values are median (dates) and mean ± SD (days).
(DOCX)

S1 Dataset Filtered daily positions derived from geolocation light logs according to individual. (XLSX)

S2 Dataset Total time spent foraging per day according to individual. (XLSX)
