# Supplementary material for: Seasonal Variation in Parental Care Drives Sex-Specific Foraging by a Monomorphic Seabird
Source: PLoS One. 2015 Nov 17;10(11):e0141190. doi: 10.1371/journal.pone.0141190 (PMC4648532; doi:10.1371/journal.pone.0141190)
Supplement: S1 Table — Letter subscripts provide additional information. Values are median (dates) and mean ± SD (days). (DOCX) [file pone.0141190.s001.docx]

**Supporting Information S1**

**S1 Table**. **Summary information for dive data according to individual and sex, showing the start and end dates for each life history stage.** Letter subscripts provide additional information. Values are median (dates) and mean ± SD (days).

| **Sex** | **Id** | **BPC** | | | **MCO** | | | **NPC** | | | **Total Days** |
| --- | --- | --- | --- | --- | --- | --- | --- | --- | --- | --- | --- |
|  |  | **Start date** | **End date** | **Total days** | **Start date** | **End date** | **Total days** | **Start date** | **End date** | **Total days** |  |
| FEMALE (n = 14) | 1604_10 | 06-Aug | 14-Aug | 9 | 15-Aug | 13-Oct | 60 | 01-Nov | 30-Jan | 91 | 160 |
|  | 1613_10 | 05-Aug | 14-Aug | 10 | 15-Aug | 13-Oct | 60 | 01-Nov | 31-Jan | 92 | 162 |
|  | 1595_11 | 03-Aug | 09-Aug | 7 | 10-Aug | 23-Sep | 45 | NA | NA | 0 | 52 |
|  | 1883_11 | 13-Aug | 17-Aug | 5 | 18-Aug | 16-Oct | 60 | 01-Nov | 27-Jan | 88 | 153 |
|  | 1885_11 | 12-Aug | 12-Aug | 1 | 13-Aug | 12-Oct | 60 | 01-Nov | 12-Jan | 73 | 134 |
|  | 1895_11 | 12-Aug | 17-Aug | 6 | 18-Aug | 16-Oct | 44 | 01-Nov | 30-Jan | 91 | 141 |
|  | 2322_12 | 22-Jul | 04-Aug | 14 | 05-Aug | 04-Oct | 60 | 01-Nov | 31-Jan | 92 | 166 |
|  | 2475_12 | 23-Jul | 02-Aug | 11 | 03-Aug | 01-Oct | 60 | 01-Nov | 31-Jan | 92 | 163 |
|  | 2475_13 | 17-Jul | 25-Jul^C^ | 9 | 26-Jul | 23-Sep | 60 | 01-Nov | 01 Dec | 31 | 100 |
|  | 2852_14 | NA ^WS^ | NA ^WS^ | NA ^WS^ | NA ^WS^ | NA ^WS^ | NA ^WS^ | 01-Nov | 31-Jan | 91 | 91 |
|  | 2857_14 | NA ^WS^ | NA ^WS^ | NA ^WS^ | NA ^WS^ | NA ^WS^ | NA ^WS^ | 02-Nov | 25-Jan | 85 | 85 |
|  | 2861_14 | NA ^WS^ | NA ^WS^ | NA ^WS^ | NA ^WS^ | NA ^WS^ | NA ^WS^ | 03-Nov | 31-Jan | 90 | 90 |
|  | 2862_14 | 23-Jul | 26-Jul | 4 | 27-Jul | 24-Sep | 60 | 01-Nov | 07-Jan | 68 | 132 |
|  | 2889_14 | 01-Aug | 01-Aug | 1 | 31-Jul | 29-Sep | 60 | NA | NA | 0 | 66 |
| **Summary** | | **03-Aug** | **09-Aug** | **7 ± 4.1^1^**  **(n = 11)** | **10-Aug** | **04-Oct** | **57 ± 6.9^1^**  **(n = 11)** | **01-Nov** | **30-Jan** | **82 ± 17.9^1^**  **^(n = 12)^** | **121.1^1^ ± 39.3** |
| MALE (n = 8) | 1470_10 | 08-Aug | 15-Aug | 8 | 16-Aug | 10-Oct | 56 | 01-Nov | 02-Jan | 63 | 127 |
|  | 1889_11 | 07-Aug | 13-Aug | 7 | 14-Aug | 22-Oct | 70 | 01-Nov | 16-Dec | 46 | 123 |
|  | 1891_11^UM^ | 07-Aug | 10-Aug | 4 | 11-Aug ^UM^ | 09-Oct ^UM^ | 60 ^UM^ | 01-Nov | 31-Jan | 92 | 156 |
|  | 1894_11 | 14-Aug | 16-Aug | 3 | 17-Aug | 15-Oct | 60 | 01-Nov | 31-Jan | 92 | 155 |
|  | 1894_12 | 28-Jul | 30-Jul | 3 | 31-Jul | 13-Oct | 75 | NA | NA | 0 | 78 |
|  | 2322_13 | 17-Jul | 24-Jul | 8 | 25-Jul | 27-Sep | 65 | 01-Nov | 29-Dec | 59 | 132 |
|  | 2854_14 | NA ^WS^ | NA ^WS^ | NA ^WS^ | NA ^WS^ | NA ^WS^ | NA ^WS^ | 01-Nov | 31-Jan | 91 | 91 |
|  | 2855_14 | 27- Jul | 27-Jul ^ACT^ | 1 | 28-Jul | 16-Sep | 51 | 01-Nov | 25-Nov | 25 | 77 |
| **Summary** | | **07-Aug** | **10-Aug ±** | **4.9 ± 2.8^1^**  **(n = 7)** | **07-Aug** | **10-Oct** | **62.8 ± 8.9^1^**  **(n = 6)** | **01-Nov** | **31-Jan** | **67 ± 26.2^1^**  **(n = 7)** | **117.4^1^ ± 31.9** |

^C^ different from colony departure since partner left with chick on 25 Jul, ^UM^ unsuccessful male [excluded during MOC], ^WS^ winter-spring (log starts in November), ^ACT^ departed colony 2 days earlier, **^1^** subscripts will be different if M ≠ F (t.test).
